# Mortuary Statistics

**Published:** 1880-11

**Authors:** 


### SEPT'R REPORT OF DEATHS AND INTERMENTS IN BALTIMORE.

| CAUSE OF DEATH. | No. | CAUSE OF DEATH. | No. | CAUSE OF DEATH. | No. |
|---|---|---|---|---|---|
| Abscess, Hip | 1 | Dropsy, (Gen'l) | 2 | Marasmus | 21 |
| " Psoas | 1 | Drowned | 6 | Measles | 1 |
| Albumensarea | 2 | Dysentery | 1 | Metritis | 1 |
| Aneurism of Aorta | 1 | Entero Colitis | 2 | Meningitis | 5 |
| Apoplexy | 12 | Epilepsy | 2 | " Tubercular | 11 |
| Asphyxia | 2 | Erysipelas | 2 | " Cerebro Spinal | 3 |
| Asthma | 4 | Fever, Bilious | 1 | Murder | 2 |
| Asthenia | 4 | " Congestive | 1 | Nervous Prostration | 1 |
| Bronchial Catarrh | 1 | " Intermittent | 1 | Necrosis | 1 |
| Bright's Disease | 5 | " Malarial | 5 | Neuralgia of Heart | 1 |
| Burns | 2 | " Puerperal | 1 | Old Age | 21 |
| Capillary Bronchitis | 1 | " Pernicious | 1 | Potts Disease | 1 |
| Cancer, Axillary Glands | 1 | " Remittent | 2 | Paralysis | 10 |
| " Breast | 3 | " Scarlet | 24 | Pneumonia | 17 |
| " Liver | | " Typhoid | 26 | Premature Birth | 10 |
| " Uterus | 5 | " Typho-Malarial | 6 | Pyæmia | 1 |
| " Stomach | 4 | Gangrene Senile | 2 | Rheumatism, Inflammatory | 2 |
| Cynanche Trachealis | 1 | Gastro Enteritis | 4 | Scrofula | 2 |
| Child Birth | 2 | Hemorrhage, Bowels | 1 | Spina Bifida | 1 |
| Cholera Infantum | 26 | " Umbilical | 1 | Softening of Brain | 2 |
| " Morbus | 1 | " Brain | 1 | Stricture Intestines | 3 |
| Concussion of Brain | 1 | Hydrocephalus | 5 | Sun Stroke | 1 |
| Congestion of Brain | 4 | Inanition | 17 | Suicide | 3 |
| " Liver | 1 | Insanity | 1 | Syphilis | 2 |
| " Lungs | 2 | Inflammation of Brain | 1 | Tabes Mesenterica | 2 |
| Consumption of Lungs | 111 | " Bladder | 1 | Tetanus | 2 |
| Convulsions | 19 | " Bowels | 5 | Trismus Nascentium | 3 |
| " Puerperal | 1 | " Stomach | 2 | Tumor, Stomach | 1 |
| Compression of Brain | 2 | " Bronchi | 1 | " Ovarian | 1 |
| " Spinal Cord | 1 | " Liver | 4 | Unknown, Adult | 1 |
| Croup | 7 | " Peritoneum | 3 | Unknown Infantile | 5 |
| Cyanosis | 2 | " Tonsils | 1 | Whooping Cough | 19 |
| Congestive Chill | 1 | Intussusception | 2 | Wounds | 5 |
| Dentition | 13 | Intemperance | 3 | Winslow's Soothiug Syrup | 1 |
| Diarrhœa | 14 | Jaundice | 2 | Non Viable | 1 |
| Diphtheria | 20 | Malformation | 2 | | |
| Disease of Heart | 11 | Mal-nutrition | 4 | Total | 598 |

THE DEATHS REGISTERED IN THE MONTH INCLUDE

| | | | |
|---|---|---|---|
| Under 1 year | 145 | Between 20 and 30 " | 72 |
| Between 1 and 2 years | 60 | " 30 and 40 " | 44 |
| " 2 and 5 years | 35 | " 40 and 50 " | 40 |
| | | " 50 and 60 " | 40 |
| Under 5 years | 240 | " 60 and 70 " | 38 |
| Between 5 and 10 years | 44 | " 70 and 80 " | 26 |
| " 10 and 15 " | 11 | " 80 and 90 " | 17 |
| " 15 and 20 " | 22 | " 90 and 100 " | 4 |

NATIVITY, ETC.

| | |
|---|---|
| United States—White | 355 |
| " Colored | 139 |
| Foreign | 104 |
| Total for the month | 598 |

Among the decedents were 51 above the age of 70 years, whose combined ages were 4,020 years, averaging 79 years.

Estimated Population, 393,796. White, 338,384. Colored, 55,412. Annual Death Rate during the month, whole population, per 1,000—18.20. White, 16,27. Colored, 30.32.

By Order: JAMES A. STEUART, M. D., Commissioner of Health and Registrar.

A. R. CARTER, Secretary.

---

U. S. SIGNAL OFFICE, BALTIMORE, MD., October 1, 1880.

### METEOROLOGICAL SUMMARY FOR THE MONTH OF SEPTEMBER, 1880.

#### PRESSURE.

Mean Monthly Barometer, - - - - - 30.075 inches.
" " " for past 10 years, - - 30.092 "
Highest Barometer, - - - - - - 30.403 " on the 11th.
Lowest " - - - - - - - 29.681 " on the 28th.
Monthly Range of Barometer, - - - - - .722 "

#### TEMPERATURE.

Mean Monthly Thermometer, - - - - - 68.4 degrees.
" " " for past 10 years, - 67.3 "
Highest Thermometer, - - 91 degrees on 4th. Monthly Range, - - 91 degrees.
Lowest " - - - 50 " on 30th. Mean Daily Range, - - 15.5 " .

#### WIND, ETC.

Prevailing Direction of Wind, Northwest. Total Amount of Rainfall, 1.78 inches.
Mean Velocity of Wind, 5 miles per hour. Number of Days on which Rain fell, 9.
Highest Velocity of Wind, 17 " " on 30th. Mean Relative Humidity, 66.1 per cent.
Total Movement of Wind, 3,629 miles.

ROBERT SEYBOTH, in charge.

